# Endoskopische Ohrchirurgie – mehr als eine Ergänzung zum Mikroskop?

**DOI:** 10.1007/s00106-021-01105-1

**Published:** 2021-09-27

**Authors:** Serena Preyer, Parwis Agha-Mir-Salim

**Affiliations:** 1 Klinik für Hals-Nasen-Ohren-Heilkunde, Kopf- und Halschirurgie und plastische Gesichtschirurgie, Ohrenschwerpunkt Karlsruhe, ViDia-Kliniken Karlsruhe, Steinhäuserstraße 18, 76133 Karlsruhe, Deutschland; 2 Klinik für Hals‑, Nasen-, und Ohrenheilkunde, Kopf- und Halschirurgie, Plastische Operationen, Zentrum für Hörimplantate, Vivantes Hörzentrum Berlin (HZB) Vivantes Klinikum im Friedrichshain, Landsberger Allee 49, 10249 Berlin, Deutschland

Liebe Leserinnen und Leser,

Endoskope haben seit der Erfindung des „Lichtleiters“ durch Philipp Bozzini im Jahr 1806 in der Medizin ihren festen Platz zur Visualisierung von Hohlräumen. Die stetige Weiterentwicklung dieser Systeme durch Georg Wolf 1911, Karl Storz 1945 und Harold Hopkins 1961 bis hin zur heutigen digitalen Kameratechnik ermöglichen einen breiten diagnostischen, aber auch chirurgischen Einsatz auf verschiedenen Gebieten der Medizin. In der Hals-Nasen-Ohren-Heilkunde hat sich die endoskopische Technik vor allem in der Nasennebenhöhlenchirurgie seit den 1980er-Jahren fest etabliert.

Die moderne Ohrchirurgie wurde im letzten Jahrhundert erst durch die Einführung des binokularen Operationsmikroskops möglich. Die Verwendung des Mikroskops hat jedoch auch Grenzen. Endoskope wurden daher schon im letzten Jahrhundert z. B. von Wullstein als Ergänzung zur klassischen Ohrmikroskopie und Erweiterung ihrer Möglichkeiten geprüft und ihr Einsatz erstmals im Jahr 1982 durch Nomura und 1990 von Takahashi beschrieben. Das Einsatzgebiet beschränkte sich zunächst auf das Mittelohr und die Tuba Eustachii. Die Möglichkeit eines breiteren Einsatzes des Endoskops auch zur Entfernung von Cholesteatomen wurde unter anderem von Thomassin 1990 und Yung 1994 erkannt. In den vergangenen 15 Jahren hat diese Technik international eine rasante Entwicklung unter anderem durch die Arbeitsgruppen um Nogueira, Presutti, Ayache, Tarabichi, Lee und Cohen, Kozin und viele mehr erfahren. Sie konnten zeigen, dass die endoskopische Ohrchirurgie (EES) eine Reihe von Vorteilen bei vergleichbaren Ergebnissen bieten kann und breit einsetzbar ist. Insbesondere eröffnet das Endoskop die Möglichkeit des minimal-invasiven Vorgehens durch den äußeren Gehörgang bei komplexer Mittelohrchirurgie.

Das Endoskop hat durch die Anwendung von Winkeloptiken Vorteile

In Deutschland wird Mittelohrchirurgie meist klassisch als mikroskopischer Eingriff durchgeführt. Passend zur internationalen Entwicklung fand die EES in den letzten 10 Jahren zwar zunehmend Beachtung, insgesamt jedoch erscheint diese Technik in Deutschland in der Ohrchirurgie bei weitem nicht so etabliert wie in der funktionellen Nasennebenhöhlenchirurgie. Die Argumentation gegen die Verwendung des Endoskops im Mittelohr erinnert sehr an die Diskussionen, die bei der Einführung der Functional Endoscopic Sinus Surgery (FESS) geführt wurden [1]. Gerade aus der Nasennebenhöhlenchirurgie sind jedoch die großen Vorteile des Endoskops beispielsweise durch die Anwendung von Winkeloptiken hinlänglich bekannt.

Analog hierzu bietet das Endoskop im Mittelohr und an der lateralen Schädelbasis zusätzliche Informationen und Möglichkeiten und kann das Mikroskop an vielen Stellen ergänzen.

In dem vorliegenden Sonderheft werden einige Aspekte des endoskopischen Operierens am Ohr beleuchtet. Es erfolgt eine Bestandsaufnahme der endoskopischen Ohrchirurgie in Deutschland durch *P. Mir-Salim*. *Adrian James* zeigt seine Ergebnisse bei der Tympanoplastik an pädiatrischen Patienten. *A. Bozzato* und *V. Flockerzi* beleuchten in ihrem Artikel die Möglichkeiten und Grenzen der endoskopischen Ossikuloplastik. *Fernandez* und *Presutti* analysieren eindrucksvoll die Grenzen dieser Operationstechnik. In einer Arbeit zu den Operationszeiten bei der endoskopischen Ohrchirurgie analysiert *S. Preyer* die Praktikabilität in einem deutschen Kliniksetting. In den Arbeiten werden die Vor- und Nachteile der endoskopischen Ohrchirurgie aufgezeigt.

Beide Autoren dieses Editorials wurden mit dem Mikroskop in der Ohrchirurgie trainiert und haben Erfahrung mit der endoskopischen Nasennebenhöhlenchirurgie. Aus eigener Erfahrung lässt sich sagen, dass die sterile Abdeckung des Mikroskops und das zusätzliche Richten des Endoskops für das OP-Personal zunächst aufwendig scheint. Es erfordert eine gewisse Umgewöhnung, dass die OP-Assistenz nicht mehr gegenüber, sondern neben dem Operateur sitzt.

Diese Anfangsschwierigkeiten sind nicht größer als bei der Einführung anderer technischer Neuerungen. Der transmeatale Zugang als präferierter Weg zum Mittelohr bei der endoskopischen Ohrchirurgie ist der direkteste Weg zum Mittelohr. Für die Patienten ist es eine Erleichterung, wenn komplexe mittelohrchirurgische Prozeduren durch den Gehörgang durchgeführt werden. Gerade bei Second-Look-Operationen wird dies von den Patienten, die beides kennen, sehr dankbar angenommen.

Für den Operateur mit Erfahrung in der mikroskopischen Ohrchirurgie ist die Lernkurve steil. Es werden etwa 50–100 Ohroperationen benötigt, bis sich Vertrautheit mit dem Handling des Endoskops im Ohr einstellt. Zu Beginn empfiehlt sich der Einsatz des Endoskops bei primär mikroskopischen Ohroperationen, um in Winkel zu schauen. Im zweiten Schritt können kleinere chirurgische Maßnahmen, wie das Legen eines Paukenröhrchens oder die Myringoplastik, ohne Auslösen des Anulus, geübt werden. Darauf aufbauend werden Eingriffe im Mittelohr geübt, bei denen aber z. B. der tympanomeatale Lappen noch mikroskopisch gehoben wird. In der Lernphase, aber auch später sollten Operateur*innen jederzeit die Möglichkeit haben und auch nicht zögern, Teilschritte einer primär endoskopisch geplanten Operation mikroskopisch durchzuführen, wenn damit ein besseres Ergebnis erreicht werden kann. Dies ist keineswegs als Scheitern anzusehen, sondern dient dem Erhalt der Qualität. Mit zunehmender Erfahrung mit der Verwendung des Endoskops wird der Einsatz des Mikroskops immer seltener. Stark blutende Operationsgebiete bleiben auch bei längerer Erfahrung in der endoskopischen Ohrchirurgie eine Herausforderung. Erkrankungen des Mastoids und Operationen mit viel Bohrarbeit sind klare Indikationen für den Einsatz des Mikroskops.

Mit dem vorliegenden Sonderheft möchten wir unseren angehenden und erfahrenen ohrchirurgischen Kolleg*innen in Deutschland Mut machen, ja sie regelrecht animieren, das Endoskop im Mittelohr noch häufiger einzusetzen. Die vielen internationalen Arbeitsgruppen haben gezeigt, dass die Möglichkeiten der ohnehin faszinierenden sanierenden und rekonstruktiven Mittelohrchirurgie hierdurch deutlich erweitert und weiterentwickelt werden können. Insbesondere die Patient*innen sind sehr dankbar für diesen minimal-invasiven, transmeatalen Zugang.

Serena Preyer

Parwis Agha-Mir-Salim

## References

[1] Kennedy DW (1986). Serious misconceptions regarding functional endoscopic sinus surgery. Laryngoscope.

